# Chemical Compositional Analysis of Catalytic Hydroconversion Products of Heishan Coal Liquefaction Residue

**DOI:** 10.1155/2017/4303596

**Published:** 2017-01-30

**Authors:** Xiaoming Yue, Yajun Wu, Shuangquan Zhang, Xiaoqin Yang, Xianyong Wei

**Affiliations:** Key Laboratory of Coal Processing and Efficient Utilization (Ministry of Education) and School of Chemical Engineering and Technology, China University of Mining and Technology, Xuzhou 221116, China

## Abstract

Liquefaction residue of Heishan bituminous coal (HLR) was subject to two hydroconversion reactions under 5 MPa initial pressure of hydrogen at 300°C for 3 h, without catalyst and with acid supported catalyst (ASC), respectively. The reaction products were analyzed with gas chromatography/mass spectrometer (GC/MS). The results show that 222 organic compounds were detected totally in the products and they can be divided into alkanes, aromatic hydrocarbons (AHCs), phenols, ketones, ethers, and other species (OSs). The yield of hydroconversion over the ASC is much higher than that without catalyst. The most abundant products are aromatic hydrocarbons in the reaction products from both catalytic and noncatalytic reactions of HLR. The yield of aromatic hydrocarbons in the reaction product from hydroconversion with the ACS is considerably higher than that from hydroconversion without a catalyst.

## 1. Introduction

Direct coal liquefaction is a significant process for transforming coal to liquid fuel and chemicals, in which considerable coal liquefaction residue (CLR) is generated. The residue obtained by the process of direct liquefaction is about 30 wt% of raw coal [1]. It is important to find a way to utilize the CLR efficiently for improving the economy of coal utilization. Even after liquefaction, CLR still retains part of organic macromolecular structure such as aromatic hydrocarbons together with the unreacted coal, the minerals from coal, and the liquefaction catalyst [2–6]. But CLR is difficult to be used due to the fact that presence of an amount of polyaromatics and specific structures of CLR are not clear at present. Obtaining more information on the molecular structure of CLR as well as getting more soluble fraction from CLR is indispensable for utilization of CLR efficiently.

Catalyst plays a very important role for cutting off the chemical bonds in the macromolecule structure of coal and solid acid is a kind of the important and efficient catalysts in direct coal liquefaction [7–9]. Shui et al. [10] found that the acidic catalyst BF_3_/SBA-15 they prepared has high hydroliquefaction of thermal dissolution soluble fractions. Other solid acids such as SO_4_^2−^/Fe_2_O_3_, SO_4_^2−^/SnO_2_, and SO_4_^2−^/Mo/Fe_2_O_3_ were used in direct coal liquefaction [11–13]. However, few reports have paid attention to the use of solid acid catalyst in CLR hydroconversion.

In this study, a kind of acid supported catalyst (ASC) was prepared and ASC-catalyzed hydroconversion of a CLR from Heishan bituminous coal was investigated.

## 2. Experimental Methods

The liquefaction residue of Heishan bituminous coal (HLR) was obtained by direct liquefaction under 19 MPa H_2_ at 455°C. The HLR was ground to <75 *μ*m, dried in vacuum at 80°C, and stored with nitrogen.  Table 1 lists the proximate and ultimate analyses of the HLR.

Solvent cyclohexane and petroleum ether (PE) were commercially purchased and then distillated using a rotary evaporator (BÜCHI Labortechnik AG, Flawil, Switzerland). Activated carbon (AC) and antimony pentachloride (SbCl_5_) were commercially available. AC was ground to <75 *μ*m, dried in vacuum at 80°C, and stored with nitrogen before use. The ASC was prepared with AC and SbCl_5_ by impregnation method under microwave.

1 g HLR, 0.4 g ASC, and cyclohexane (30 mL) were put into a stainless autoclave (60 mL volume) with magnetic stirrer. After being purged with N_2_ three times to remove air from the autoclave, the autoclave was pressurized with H_2_ to 5 MPa, then heated to 300°C at a rate of 20°C/min, and kept for 3 h. After that, in an ice bath, the autoclave was cooled rapidly. The reaction mixture which was thoroughly removed from the autoclave using PE as solvent was filtrated by a membrane filter with pore size of 0.8 *μ*m. The filter cake was thoroughly extracted with PE in a Soxhlet extractor and then dried in vacuum at 80°C for 12 h. The filtrate and PE extraction were combined and concentrated by a rotary evaporator to obtain concentrated reaction product. The form of the yield is expressed as

(1) $$\begin{array}{c} Y=\left(\;1-\frac{m_{1}-m_{c}}{m_{0}}\;\right)\times 100\%, \end{array}$$

where *m*_1_ refers to the mass of the dried filter cake (reaction residue); *m*_*c*_ is the mass of the catalyst in the reaction; *m*_0_ is the mass of HLR in the reaction.

The organic compounds in the reaction product were identified with gas chromatography/mass spectrometer (GC/MS; Hewlett-Packard Company, Hewlett-Packard 6890/5973) and quantified with gas chromatography (GC; HP 6890). Fourier transform infrared (FTIR) was collected at room temperature on a Nicolet Magna IR-560 infrared spectrometer. N_2_ adsorption-desorption isotherms were determined by an Autosorb-1-MP specific surface area and pore size analyzer at 77 K from Quantachrome Instruments Company to obtain pore volume, average pore diameter, and surface area of the AC and ASC.

As a comparison reaction, the procedure of noncatalytic hydroconversion (NCHC) is the same with the catalytic hydroconversion (CHC) but without any catalyst.

## 3. Results and Discussion

**Figure 1:**
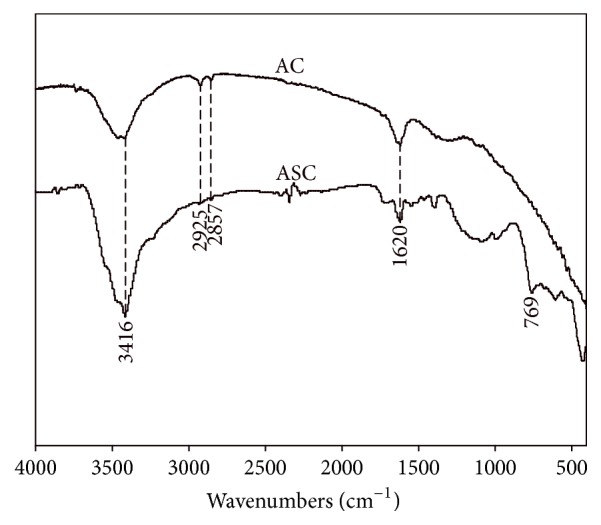
FTIR spectra of the AC and ASC.

As Figure 1 shows, the absorbances at 2925 and 2857 cm^−1^ attributed to CH_3_ group and >C-C< moiety [14] were observed in the AC, which had to be found in the ASC. The absorbances of -OH group at 3416 cm^−1^ and >C=C< moiety at 1620 cm^−1^ in the ASC are stronger than those in the AC [14]. The absorbance between 800 and 600 cm^−1^ attributed to C-Cl stretching vibration absorption [15]. The appearance of C-Cl bonds at 769 cm^−1^ in ASC indicates that the reaction of SbCl_5_ with the AC was carried out during the process of ASC preparation [16, 17]. The pore volume, average pore diameter, and surface area of AC were 0.53 cm^3^/g, 2.85 nm, and 743 m^2^/g, respectively. However, the pore volume, average pore diameter, and surface area of ASC were 0.26 cm^3^/g, 2.66 nm, and 526.3 m^2^/g, respectively. It indicates that the pore diameter of catalyst decreased after impregnation with SbCl_5_; the active component loaded on the inner surface of the pore in AC was possibly the reason. Therefore, the pore volumes, average pore diameter, and surface areas were all decreased after impregnation.

The yields of NCHC and CHC of HLR are 35.49% and 57.11%, respectively. Compared with the NCHC, the yield of CHC is remarkably improved. These data show that the ASC plays a significant role to promote the decomposition of HLR.

**Figure 2:**
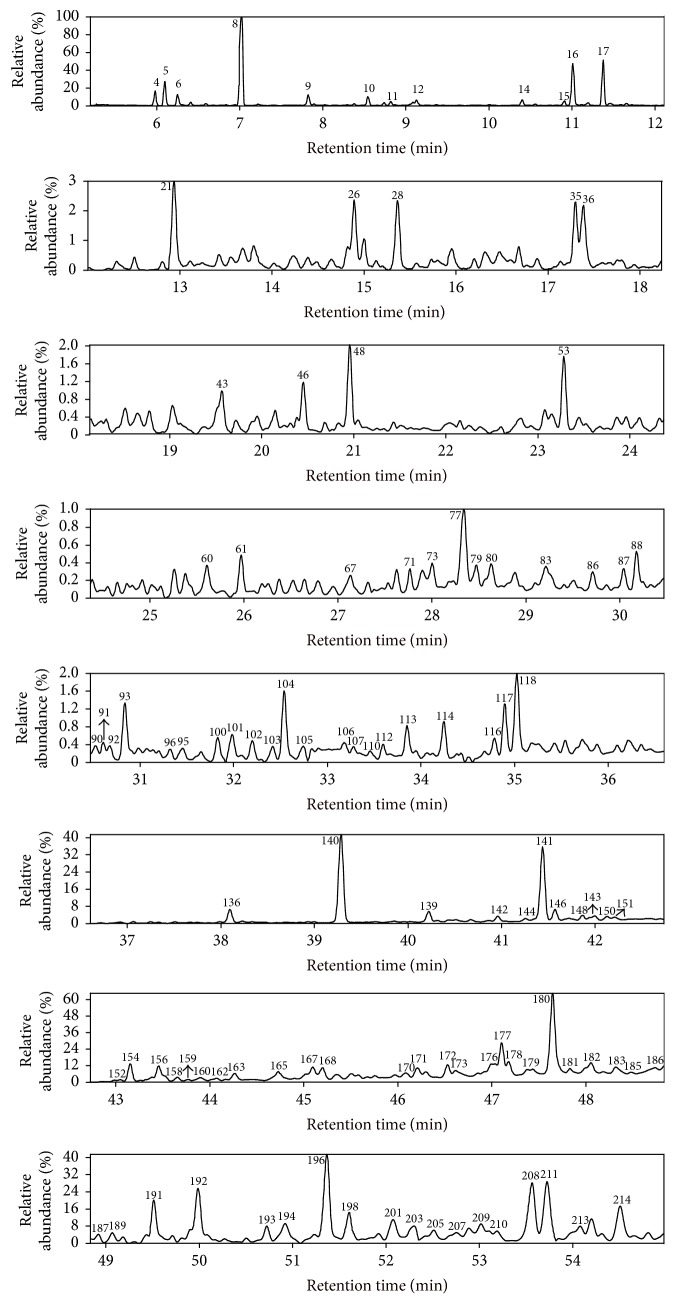
Total ion chromatogram of the reaction product from NCHC of HLR.

**Figure 3:**
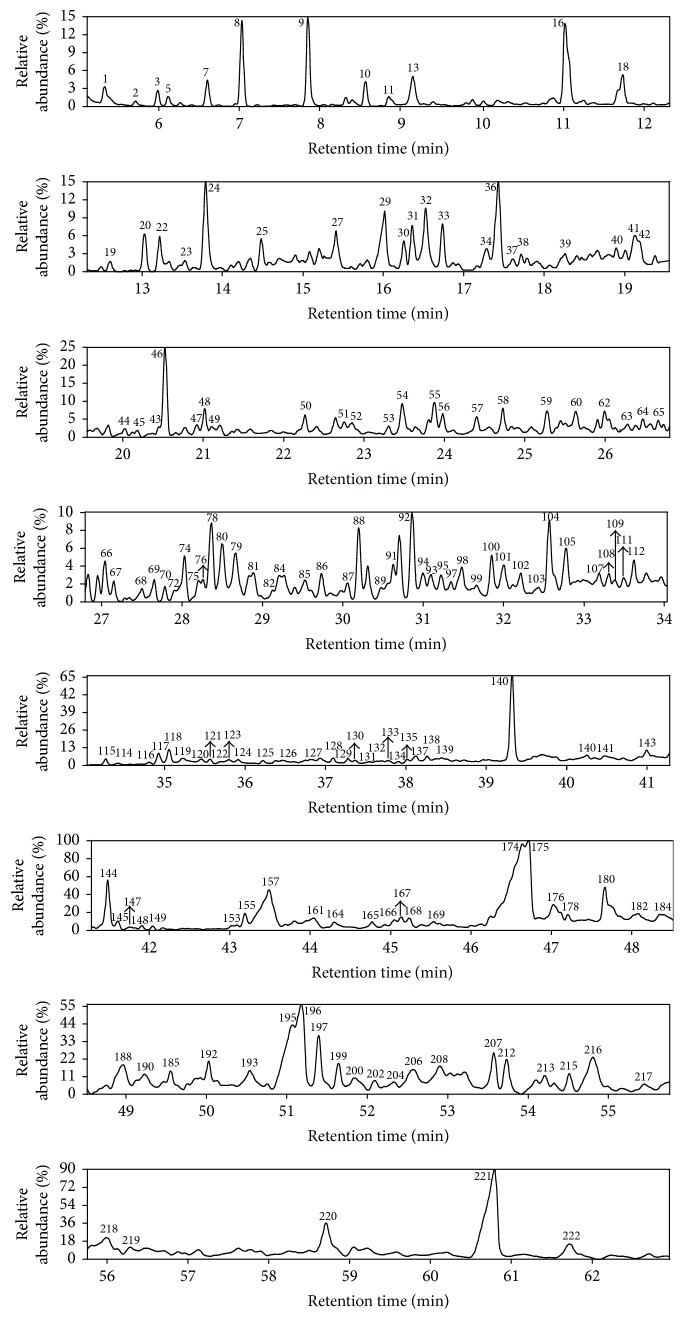
Total ion chromatogram of the reaction product from CHC of HLR.

The reaction products from NCHC and CHC of HLR are simplified as RPNC and RPC, respectively. Figures 2 and 3 exhibit the total ion chromatograms (TIC) of RPNC and RPC with GC/MS. Totally, 222 organic compounds were identified, and they can be divided into six groups: alkanes, aromatic hydrocarbons (AHCs), phenols, ketones, ethers, and other species (OSs), as listed in Tables 2 and 3 and Tables S.1–4 (see Supplementary Material available online at https://doi.org/10.1155/2017/4303596).

**Figure 4:**
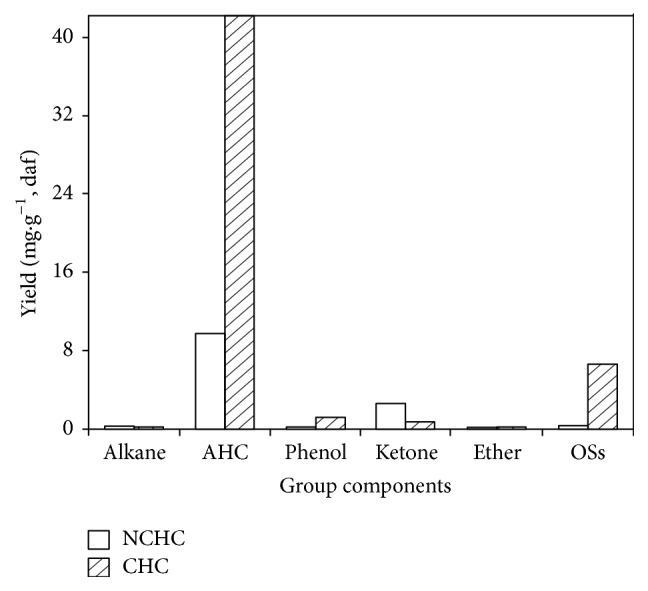
Distribution of group components in the reaction products from NCHC and CHC of HLR.

As shown in Figure 4, the yields of group components from RPNC increase in the order ethers < phenols < alkanes < OSs < ketones < AHCs, while the order of RPC is alkanes < ethers < ketones < phenols < OSs ≪ AHCs. The yield of RPNC (12.94 mg·g^−1^, daf) is much lower than that of RPC (50.91 mg·g^−1^, daf); extremely more organic compounds were determined in the RPC than those from RPNC.

As listed in Table 2, four alkanes (206.1 *μ*g·g^−1^, daf) were detected in the RPNC, including one cyclic hydrocarbon, two N-alkanes, and one branched alkane. Six alkanes (180.3 *μ*g·g^−1^, daf) were detected in the RPC, including five N-alkanes and one branched alkane, with carbon atoms number from 15 to 20. No olefin was detected. Most of the side chains in coal were removed during the coal liquefaction process; it is the possible and reasonable reason for the low yield of the alkanes.

In total, 171 AHCs were detected in the reaction products, including 81 and 130 AHCs appearing from RPNC and RPC, respectively, as shown in Table S.1 (supplementary data). The yields of AHCs are 9.7 and 42.1 mg·g^−1^ (daf) and the relative contents are 73.0% and 82.5% in the RPNC and RPC, respectively. 14 homologues of benzene, 7 homologues of fluorine, 17 homologues of naphthalene, 7 homologues of anthracene, 6 homologues of phenanthrene, and 30 condensed arenes with carbon atoms number greater than 4 were found in the RPNC. AHCs detected in the RPC include 33 homologues of benzene, 9 homologues of fluorine, 26 homologues of naphthalene, 10 homologues of anthracene, 12 homologues of phenanthrene, 4 homologues of indene, and 36 condensed arenes with carbon atoms number greater than 4. Among them, the yield of benzoperylene (peak 175) is the most and the relative content is 13.4% in the RPC of HLR. Secondly, the relative content of 2-methylbenzoperylene (peak 221) is 10.4%. The AHCs in the RPC (42.1 mg·g^−1^, daf) are remarkably higher than those in the RPNC (9.7 mg·g^−1^, daf). Our previous investigation showed that C_ar_-C_alk_ bridge bond in di(1-naphthyl)methane can be especially ruptured over ASC to afford naphthalene and 1-methylnaphthalene under 5 MPa initial pressure of hydrogen at 300°C [18, 19]. H_2_ was cleaved to H^−^ adhering on the surface of the catalyst with H^+^moving freely over the ASC with strong acidity. The addition of H^+^ to the ipso-position of an aromatic ring in the macromolecule of HLR brings about cleavage of the C_ar_-C_alk_ bridge bond, leading to the release of AHCs, which is the appropriate reason for the significantly higher yield of the RPNC. Furthermore, it can be speculated that a large number of AHCs are connected with the macromolecular skeleton of coal liquefaction residue by bridge bonds.

As exhibited in Table 3, 3 and 9 phenols were detected in the RPNC and RPC, respectively. The yields of phenols in the RPC (1193.2 *μ*g·g^−1^, daf) are dramatically higher than those in the RPNC (171.9 *μ*g·g^−1^, daf). There are many oxygen-containing functional groups in lignite and the C-O bond is a kind of the important bridge bonds linking aromatic hydrocarbons or alkanes [20]. The addition of H^+^ to the ipso-position of the phenoxy in the HLR leads to cleavage of the C-O bond to receive the phenols; it should be the main reason for significantly higher yields of phenols in the RPC compared to those from RPNC [19]. The fracture of C-O bonds is facilitated more easily with the catalysis of the ASC.

## 4. Conclusions

Organic compounds of the reaction product from RPNC and RPC detected by GC/MS include alkanes, AHCs, phenols, ketones, ethers, and OSs. The yield of CHC is obviously improved compared with the NCHC and it shows that the ASC plays a significant role to promote the decomposition of HLR. Much more AHCs and phenols were released from RPC than those from RPNC. The hydroconversion of HLR under 300°C over the ASC not only provides an efficient approach for producing lots of value-added chemicals from the residue but also provides the information of macromolecular structures of the residue.

## Supplementary Material

As listed in Table S.1, 171 AHCs were detected in the reaction products, including 81 and 130 AHCs appeared from RPNC and RPC respectively, including homologues of benzene, fluorine, naphthalene, anthracene, phenanthrene and condensed arenes. As shown in Table S.2, 10 and 3 ketones were found in the RPNC and RPC, respectively. Table S.3 listed the 2 and 8 ethers detected from RPNC and RPC, respectively. Other species organic compounds were listed in S. 4, 8 and 12 OSs were discovered in the RPNC and RPC, respectively, including alcohols, esters, sulfur-containing compounds and nitrogen-containing compound.

## Figures and Tables

**Table 1:** Proximate and ultimate analyses (*W*%) of HLR.

Proximate analysis			Ultimate analysis (daf)				
*M*_ad_	*A*_d_	*V*_daf_	C	H	N	O^a^	S
0.19	21.64	39.07	75.09	1.29	1.36	24.42	3.79

^a^By difference.

**Table 2:** Alkanes detected in the reaction product from NCHC and CHC of HLR.

Peak	Compounds	NCHC	CHC
4	Methylcyclohexane	√	
59	Pentadecane		√
70	Hexadecane		√
89	Heptadecane	√	√
106	Octadecane	√	√
107	8-Methylheptadecane	√	√
128	Eicosane		√

**Table 3:** Phenols detected in the reaction product from NCHC and CHC of HLR.

Peak	Compounds	NCHC	CHC
22	*o*-Cresol		√
24	*m*-Cresol		√
32	3-Ethylphenol		√
34	3,4-Dimethylphenol		√
40	2-Ethyl-6-methylphenol		√
47	2,3-Dihydro-1*H*-inden-5-ol		√
61	2,4,6-Triisopropylphenol	√	
118	(*E*)-4-Styrylphenol	√	√
119	(*E*)-4-Styrylphenol		√
167	Chrysen-6-ol	√	√
