# Supplementary material for: Chemical Compositional Analysis of Catalytic Hydroconversion Products of Heishan Coal Liquefaction Residue
Source: Int J Anal Chem. 2017 Jan 30;2017:4303596. doi: 10.1155/2017/4303596 (PMC5304314; doi:10.1155/2017/4303596)
Supplement: Supplementary file 1 — As listed in Table S.1, 171 AHCs were detected in the reaction products, including 81 and 130 AHCs appeared from RPNC and RPC respectively, including homologues of benzene, fluorine, naphthalene, anthracene, phenanthrene and condensed arenes. As shown in Table S.2, 10 and 3 ketones were found in the RPNC and RPC, respectively. Table S.3 listed the 2 and 8 ethers detected from RPNC and RPC, respectively. Other species organic compounds were listed in S. 4, 8 and 12 OSs were discovered in the RPNC and RPC, respectively, including alcohols, esters, sulfur-containing compounds and nitrogen-containing compound. [file 4303596.f1.doc]

**Table S.1.**  Arenes detected in the PEEF from NCHC and CHC of HBCLR.

| Peak | Compounds | NCHC | CHC | Peak | Compounds | NCHC | CHC |
| --- | --- | --- | --- | --- | --- | --- | --- |
| **1** | benzene |  | √ | **55** | 1,2-dimethylnaphthalene |  | √ |
| **7** | toluene | √ | √ | **56** | 2,7-dimethylnaphthalene |  | √ |
| **10** | m-xylene |  | √ | **57** | 2,3-dimethylnaphthalene |  | √ |
| **13** | o-Xylene | √ | √ | **58** | 1,3-dimethylnaphthalene |  | √ |
| **16** | mesitylene | √ | √ | **60** | 1,2-dihydroacenaphthylene | √ | √ |
| **18** | 1,2,3-trimethylbenzene |  | √ | **62** | 2,3,6-trimethylnaphthalene |  | √ |
| **19** | 1,2,4-trimethylbenzene |  | √ | **63** | 1-(prop-1-en-2-yl)naphthalene |  | √ |
| **20** | (E)-prop-1-enylbenzene |  | √ | **65** | 1,4,6-trimethylnaphthalene |  | √ |
| **23** | m-cymene |  | √ | **66** | 1,6,7-trimethylnaphthalene |  | √ |
| **25** | (Z)-but-1-enylbenzene |  | √ | **67** | 2,3,6-trimethylnaphthalene | √ | √ |
| **27** | 1,2,3,5-tetramethylbenzene |  | √ | **68** | 2,3,6-trimethylnaphthalene |  | √ |
| **29** | (E)-but-2-enylbenzene |  | √ | **72** | 4,4'-dimethylbiphenyl |  | √ |
| **31** | 4-methyl-2,3-dihydro-1H-indene |  | √ | **73** | 9H-fluorene | √ | √ |
| **33** | 1,2,3,4-tetrahydronaphthalene |  | √ | **74** | 2-butylnaphthalene |  | √ |
| **35** | 2-isobutyl-1,4-dimethylbenzene | √ | √ | **75** | 2-methyl-1-propylnaphthalene |  | √ |
| **36** | Naphthalene | √ | √ | **76** | 1-(prop-1-en-2-yl)naphthalene | √ |  |
| **37** | 1,6-dimethyl-2,3-dihydro-1H-indene |  | √ | **77** | 1-methylbiphenyl |  | √ |
| **38** | 2-methyl-1,2,3,4-tetrahydronaphthalene |  | √ | **78** | 2-methylbiphenyl | √ |  |
| **41** | 5,6-dimethyl-2,3-dihydro-1H-indene |  | √ | **79** | 1,2-dimethylbiphenyl |  | √ |
| **42** | 5-methyl-1,2,3,4-tetrahydronaphthalene | √ |  | **80** | 1,3'-dimethylbiphenyl | √ |  |
| **43** | 1,2,3,4,5-pentamethylbenzene |  | √ | **81** | 1,4'-dimethylbiphenyl |  | √ |
| **44** | 4,7-dimethyl-2,3-dihydro-1H-indene |  | √ | **82** | 2,3-dimethylbiphenyl |  | √ |
| **45** | 5-methyl-1,2,3,4-tetrahydronaphthalene |  | √ | **83** | ethane-1,1-diyldibenzene |  | √ |
| **46** | 1-methyl-naphthalene | √ | √ | **86** | ethane-1,1-diyldibenzene |  | √ |
| **48** | 2-methyl-naphthalene | √ | √ | **87** | 2-ethylbiphenyl | √ | √ |
| **49** | 1,4-dimethyl-1,2,3,4-tetrahydronaphthalene |  | √ | **88** | 1-ethylbiphenyl |  | √ |
| **50** | 6-ethyl-1,2,3,4-tetrahydronaphthalene |  | √ | **90** | 9,10-dihydroanthracene | √ | √ |
| **51** | Biphenyl |  | √ | **91** | 9-methyl-9,10-dihydroanthracene |  | √ |
| **52** | 1-isopropyl-3,5-dimethylbenzene |  | √ | **92** | 1,2,3,4,5,6,7,8-octahydroanthracene | √ |  |
| **54** | 2,6-dimethylnaphthalene |  | √ | **93** | 9-methyl-9H-fluorene | √ | √ |

**Table S.1.** Arenes detected in the PEEF from NCHC and CHC of HBCLR. (Continued)

| Peak | Compounds | NCHC | CHC | Peak | Compounds | NCHC | CHC |
| --- | --- | --- | --- | --- | --- | --- | --- |
| **94** | 2-methyl-9H-fluorene | √ |  | **134** | 9,10-Dimethylanthracene |  | √ |
| **95** | 1,1'-dimethylbiphenyl | √ |  | **135** | 5-phenyl-1,2,3,4-tetrahydronaphthalene |  | √ |
| **96** | 2-methyl-9H-fluorene |  | √ | **136** | 1,4-Dimethylanthracene |  | √ |
| **97** | 3,4'-dimethylbiphenyl |  | √ | **137** | 2,7-dimethylphenanthrene |  | √ |
| **98** | 4,4'-dimethylbiphenyl |  | √ | **138** | 2,5-dimethylphenanthrene |  | √ |
| **99** | 2,2'-dimethylbiphenyl |  | √ | **139** | 4,5-dihydropyrene | √ | √ |
| **100** | 2,4'-dimethylbiphenyl | √ | √ | **140** | fluoranthene |  | √ |
| **101** | 1,4'-dimethylbiphenyl | √ |  | **141** | 2-benzylnaphthalene |  | √ |
| **102** | 3-ethylbiphenyl |  | √ | **142** | pyrene | √ | √ |
| **103** | 3,3'-dimethylbiphenyl |  | √ | **143** | 2-benzylnaphthalene | √ |  |
| **104** | 1,2,3,4-tetrahydroanthracene | √ | √ | **144** | 9-allylanthracene |  | √ |
| **105** | 1,2,3,4-tetrahydrophenanthrene | √ | √ | **145** | 1-methylpyrene |  | √ |
| **108** | Phenanthrene | √ | √ | **146** | 9-butylphenanthrene | √ | √ |
| **109** | Anthracene | √ | √ | **147** | 11H-benzo[b]fluorene | √ | √ |
| **110** | 3,4'-methylenebis(methylbenzene) | √ | √ | **148** | 1-methylpyrene | √ | √ |
| **111** | 2,3-dimethyl-9H-fluorene | √ | √ | **149** | 9-allylanthracene | √ |  |
| **112** | (E)-prop-1-ene-1,2-diyldibenzene |  | √ | **150** | 1-benzylnaphthalene |  | √ |
| **113** | (E)-1-methyl-3-styrylbenzene |  | √ | **151** | 4-methylpyrene | √ | √ |
| **114** | 2-methyl-9,10-dihydroanthracene | √ |  | **152** | 2-methylpyrene | √ | √ |
| **115** | 9,9-dimethyl-9H-fluorene |  | √ | **153** | 1,4-dimethyl-5-phenylnaphthalene | √ |  |
| **121** | 9-methylanthracene | √ | √ | **154** | 1-methyl-4-p-tolylnaphthalene | √ |  |
| **122** | 2-methylphenanthrene | √ | √ | **155** | o-terphenyl | √ |  |
| **123** | 1-methylanthracene |  | √ | **156** | 1,3-dimethylpyrene |  | √ |
| **124** | 1-methylphenanthrene |  | √ | **157** | m-terphenyl | √ |  |
| **129** | 1-phenylnaphthalene |  | √ | **158** | 5,12-dihydrotetracene |  | √ |
| **130** | 9-ethylphenanthrene |  | √ | **159** | 1,3-dimethylpyrene | √ |  |
| **131** | 4,5-dimethylphenanthrene |  | √ | **160** | indeno[1,2,3-cd]pyrene |  | √ |
| **132** | 3,6-dimethylphenanthrene |  | √ | **161** | 1,4-dimethyl-2-phenylnaphthalene | √ |  |
| **133** | 1,7-dimethylphenanthrene |  | √ | **162** | 1,9-dimethylpyrene | √ |  |

**Table S.1.** Arenes detected in the PEEF from NCHC and CHC of HBCLR. (Continued)

| Peak | Compounds | NCHC | CHC | Peak | Compounds | | NCHC | CHC |
| --- | --- | --- | --- | --- | --- | --- | --- | --- |
| **163** | (E)-2-styrylnaphthalene | √ |  | **193** | 7,12-dimethyltetraphene | |  | √ |
| **164** | benzo[f]tetraphene | √ | √ | **194** | benzo[e]acephenanthrylene | | √ |  |
| **165** | 1,2,3,4-tetrahydrochrysene | √ |  | **195** | benz[e]fluorene | |  | √ |
| **166** | 1,2,3,4-tetrahydrotriphenylene |  | √ | **196** | 7,8,9,10-tetrahydrobenzo[pqr]tetraphene | |  | √ |
| **168** | chrysene |  | √ | **197** | 7,8,9,10-benzo[pqr]tetraphene | | √ |  |
| **169** | (E)-2-(1-phenylprop-1-en-2-yl)naphthalene | √ | √ | **198** | 9-benzylphenanthrene | | √ |  |
| **170** | triphenylene | √ | √ | **201** | benzo[e]pyrene | | √ | √ |
| **172** | 4-methyltetraphene | √ |  | **202** | benzo[pqr]tetraphene | | √ |  |
| **173** | 4-methylchrysene | √ |  | **203** | benzo[a]pyrene | |  | √ |
| **175** | benzo[ghi]perylene | √ | √ | **205** | 2,2'-dimethyl-1,1'-binaphthyl | | √ | √ |
| **176** | naphtho[7,8,1,2,3-nopqr]tetraphene |  | √ | **206** | 11H-indeno[2,1-a]phenanthrene | | √ |  |
| **177** | 1-methyltetraphene | √ | √ | **207** | 3-methylperylene | |  | √ |
| **178** | dinaphthalen-1-ylmethane | √ |  | **208** | | 2-benzylfluoranthene | √ | √ |
| **179** | 7-methyltetraphene | √ | √ | **209** | | 4-methylbenzo[ghi]perylene | √ | √ |
| **180** | 1,2'-methylenedinaphthalene | √ |  | **210** | | 5,6-dihydrobenzo[k]tetraphene | √ |  |
| **181** | 3-methylbenzo[c]phenanthrene | √ | √ | **211** | | 13H-dibenzo[a,h]fluorene | √ |  |
| **182** | 4-methylchrysene | √ |  | **212** | | 3-methylperylene | √ | √ |
| **183** | 1,12-dimethyltetraphene | √ |  | **213** | | 1-methylperylene | √ |  |
| **184** | 2,7-dimethyltetraphene |  | √ | **214** | | 13H-dibenzo[a,h]fluorene |  | √ |
| **185** | 7,12-dimethyltetraphene | √ |  | **215** | | picene | √ | √ |
| **186** | 1-benzylpyrene |  | √ | **216** | | 3-methylcyclopenta[ij]tetraphene |  | √ |
| **187** | 7,12-dimethyltetraphene | √ |  | **217** | | 1,2,5,6-tetrahydrocoronene |  | √ |
| **188** | 1,12-dimethyltetraphene | √ |  | **218** | | 5,6-dihydrobenzo[k]tetraphene |  | √ |
| **189** | 4-methylbenzo[ghi]perylene | √ | √ | **220** | | benzo[ghi]perylene |  | √ |
| **190** | 5,8-dimethylbenzo[c]phenanthrene | √ |  | **221** | | 2-methylbenzo[ghi]perylene |  | √ |
| **191** | 2-benzylfluoranthene |  | √ | **222** | | 4-methylbenzo[ghi]perylene |  | √ |
| **192** | 5-ethylchrysene | √ |  |  |  | |  |  |

**Table S.2.** Ketones detected in the PEEF from NCHC and CHC of HLR

| Peak | Compounds | NCHC | CHC | Peak | Compounds | NCHC | CHC |
| --- | --- | --- | --- | --- | --- | --- | --- |
| **5** | 4-methylpentan-2-one | √ | √ | **15** | 2,6-dimethylheptan-4-one | √ |  |
| **6** | 4-methylpent-4-en-2-one | √ |  | **21** | 3,3,5-trimethylcyclohexanone | √ |  |
| **8** | (E)-hex-3-en-2-one | √ | √ | **26** | 2,6-dimethylhepta-2,5-dien-4-one | √ |  |
| **9** | 4-hydroxy-4-methylpentan-2-one | √ | √ | **28** | 3,5,5-trimethylcyclohex-2-enone | √ |  |
| **12** | cyclohexanone | √ |  | **171** | 1-(pyren-1-yl)ethanone |  | √ |

**Table S.3.**  Ethers detected in the PEEF from NCHC and CHC of HLR

| Peak | Compounds | NCHC | CHC | Peak | Compounds | NCHC | CHC |
| --- | --- | --- | --- | --- | --- | --- | --- |
| **2** | 2,5-dimethylfuran |  | √ | **69** | 2-ethoxynaphthalene |  | √ |
| **3** | 1,2-diethoxyethane |  | √ | **125** | 9-methoxyanthracene |  | √ |
| **53** | oxydibenzene | √ | √ | **174** | 5-methoxybenzo[c]phenanthrene | √ |  |
| **64** | dibenzo[b,d]furan |  | √ |  |  |  |  |

**Table S.4.**  OSs detected in the PEEF from NCHC and CHC of HLR

| Peak | Compounds | NCHC | CHC | Peak | Compounds | NCHC | CHC |
| --- | --- | --- | --- | --- | --- | --- | --- |
| **11** | cyclohexanol | √ | √ | **117** | dibutyl phthalate |  | √ |
| **14** | cyclohexanethiol | √ |  | **120** | methyl palmitate |  | √ |
| **17** | 1,3-dimethyltrisulfane | √ |  | **126** | 9-methyl-9H-carbazole |  | √ |
| **30** | N-ethyl-2-methylaniline |  | √ | **127** | 3-methyl-9H-carbazole |  | √ |
| **39** | N,2,4,6-tetramethylaniline |  | √ | **199** | benzo[1,2-b:3,4-b']bis[1]benzothiophene |  | √ |
| **71** | diethyl phthalate | √ |  | **200** | benzo[1,2-b:4,5-b']bis[1]benzothiophene |  | √ |
| **84** | 3,3,4,7-tetramethylindolin-2-one | √ |  | **204** | benzo[1,2-b:5,4-b']bis[1]benzothiophene |  | √ |
| **85** | 9H-fluoren-9-ol |  | √ | **219** | dibenzo[a,c]phenazine |  | √ |
| **116** | 9H-carbazole | √ | √ |  |  |  |  |
